# Sex-Differences of Face Coding: Evidence from Larger Right Hemispheric M170 in Men and Dipole Source Modelling

**DOI:** 10.1371/journal.pone.0069107

**Published:** 2013-07-09

**Authors:** Hannes O. Tiedt, Joachim E. Weber, Alfred Pauls, Klaus M. Beier, Andreas Lueschow

**Affiliations:** 1 Department of Neurology, Charité – Universitätsmedizin Berlin, Campus Benjamin Franklin, Berlin, Germany; 2 Institute of Sexology and Sexual Medicine, Charité – Universitätsmedizin Berlin, Berlin, Germany; University of British Columbia, Canada

## Abstract

The processing of faces relies on a specialized neural system comprising bilateral cortical structures with a dominance of the right hemisphere. However, due to inconsistencies of earlier findings as well as more recent results such functional lateralization has become a topic of discussion. In particular, studies employing behavioural tasks and electrophysiological methods indicate a dominance of the right hemisphere during face perception only in men whereas women exhibit symmetric and bilateral face processing. The aim of this study was to further investigate such sex differences in hemispheric processing of personally familiar and opposite-sex faces using whole-head magnetoencephalography (MEG). We found a right-lateralized M170-component in occipito-temporal sensor clusters in men as opposed to a bilateral response in women. Furthermore, the same pattern was obtained in performing dipole localization and determining dipole strength in the M170-timewindow. These results suggest asymmetric involvement of face-responsive neural structures in men and allow to ascribe this asymmetry to the fusiform gyrus. This specifies findings from previous investigations employing event-related potentials (ERP) and LORETA reconstruction methods yielding rather extended bilateral activations showing left asymmetry in women and right lateralization in men. We discuss our finding of an asymmetric fusiform activation pattern in men in terms of holistic face processing during face evaluation and sex differences with regard to visual strategies in general and interest for opposite faces in special. Taken together the pattern of hemispheric specialization observed here yields new insights into sex differences in face perception and entails further questions about interactions between biological sex, psychological gender and influences that might be stimulus-driven or task dependent.

## Introduction

The highly specialized skill of face perception in humans is a function of a bilaterally organized neural system [1] with a supposed dominance of the right hemisphere [2] which emerges during development of cognitive functions [3]. The processing of faces involves occipito-temporal regions and most notably the fusiform gyrus (FG) which is consistently found to respond specifically to faces (for a review and argumentation in favour of domain-specific mechanisms underlying face-recognition see [4]) and may function as an entry node to subsequent processing in a more widespread cortical network [5], [6]. However, recent investigations indicate that hemispheric asymmetries in face processing may at least be partially due to a higher degree of functional lateralisation in men compared to women [7]. Analogous findings have been obtained for visuospatial abilities, e.g. mental rotation [8] and language functions [9]. In particular, studies on face processing employing behavioural tasks such as chimeric face perception or presentation of faces selectively to the right or left visual field demonstrate a higher degree of functional lateralisation to the right hemisphere in men with a more bilateral ability in women [10], [11]. ERP-studies found an increased amplitude of the face-specific N170-component in the right hemisphere only in male subjects with no such laterality effects in women [7]. The N170-response reflects structural encoding of faces [12] and in most instances correlates with the N200-component in intracranial recordings [13] and the M170-component in MEG-recordings [14], [15]. Although sometimes seen as the otherwise equivalent magnetic counterpart of the N170-component there are a few marked differences concerning the M170 due to differential sensitivity to source orientation of EEG and MEG [16], [17]. Thus, the M170 has been found to originate primarily from sources within the FG [15], [18] whereas the N170 is to a larger extent influenced by sources in other face-responsive regions, e.g. in the superior temporal sulcus (STS) [19]. The FG contains the functionally defined so called fusiform face area (FFA) which is found to be specifically engaged by face-stimuli [2].

Here, we used whole-head-MEG to study whether sex-differences in face processing are reflected in early face-specific event-related magnetic fields (ERFs), i.e. the M100 [14] and M170 [15].

## Materials and Methods

### Subjects

Twenty-six adult subjects (13 females; mean age = 25.46 years, Range 20–35) who were right-handed by self-report participated in the study. All participants had normal or corrected to normal vision and none had a history of neurological, psychiatric or any other relevant disorders. They did not have metallic implants or any other ferromagnetic objects on them. All participants gave their informed written consent prior to the experiment. The study was approved by the Local Ethics Committee (Ethikausschuss Campus Benjamin Franklin, Charité – Universitätsmedizin, Berlin).

### Stimuli and Procedure

The participants provided digital photographs displaying opposite-sex faces of personally familiar persons (partner and close friends). Out of the provided photographs 3 pictures of each category (partner, 2 friends) were singled out and processed to fit the requirements of the experiments; the faces of the persons were cut out and only pictures displaying frontal views of faces lacking possibly distracting features such as unique hairdressing, glasses etc. were selected. All faces had either neutral or positive expressions without differences between face-categories or gender. The pictures were arranged to a size of 100×100 pixels and projected on a screen in front of the subjects with a viewing angle of 11.5°. The MEG-measurements were conducted in a magnetically shielded room using a whole-head (Eagle Technology™, ET160) employing 93 first order gradiometers with a baseline of 5 cm. The experimental procedure consisted of a passive viewing paradigm; all subjects were instructed to avoid head and eye movements and to view the pictures and simultaneously imagine a comfortable situation. All faces were presented 30 times each in a randomized order with variable interstimulus intervals and for 6000 ms. The purpose of including emotional imagery and using lengthier presentation times was to study late and sustained shifts of magnetic activity related to the late positive potential (LPP) in ERP-studies. This will be reported elsewhere (Tiedt et al., in preparation). Here, the analysis is restricted to early and face-specific ERFs (M100 and M170) occurring within 200 ms as after stimulus onset.

### MEG Data Analyses

MEG-signals were digitized with a sampling rate of 500 Hertz (Hz) and a high pass filter of 0, 1 Hz and low pass filter of 200 Hz. Offline, the data were down-sampled to 250 Hz and bandpass-filtered between 0.1 and 40 Hz. To remove eye artefacts (EOG) in the data and artefacts caused by the magnetic field of the heart, the MEG data were submitted to an Independent Component Analysis (ICA) before averaging [20]. In addition to the ICA correction, EOG-artefact contaminated epochs were excluded in the conventional fashion using BESA™ (*Brain Electrical Source Analysis, MEGIS Software*). Averages of event-related fields (ERFs) were calculated over 6000 ms including a pre-stimulus baseline of 300 ms. We used automated algorithms to identify sensors detecting the M170-component (defined as a peak occurring between 140–200 ms) within a region-of-interest previously defined based on physiological assumptions comprising 26 sensors over each hemisphere covering occipito-temporal regions. A sensor-cluster including 3–8 regionally arranged sensors exhibiting at least 50%, ideally 70% of the maximum peak amplitude was selected for further analysis. For further comparisons positive peaks (M170) were inverted by multiplicating the values with (–1) to match polarities between hemispheres and subjects. A peak preceding the M170-response between 70–130 ms was defined as the M100-component. Peak-amplitude und -latency were analyzed for both the M100 and M170 in each hemisphere. Furthermore, individual dipole localizations and strength of the M170 were calculated with the program Brain Electrical Source Analysis (BESA 2000™, MEGIS Software). The dipole fit time intervals were selected to include a time-window of 40 ms around the M170 in both hemispheres, resulting in an average time-window of 146–186 ms. The M170 was modelled by a single dipole in each hemisphere using a single-layer spherical head model and after introducing a symmetry constraint. In a “fixed” condition dipole strength within this time-window was determined based on Talairach coordinates of the FG (x = ±29.0, y = −62.0, z = −15.0) obtained in a previous investigation on source localization of the N170/M170-response [18]. Furthermore, in a “free” condition without preset coordinates dipole strength in each hemisphere as well as Talairach coordinates of the underlying source localization were determined. However, we could not obtain a plausible solution in 2 subjects using this “free” algorithm; in these cases the dipoles were located outside the head or both at midline.

### Statistical Analysis

Normal distribution of the data was established using the Kolmogorov-Smirnov-Test. Amplitude and latency of M100 and M170 were analyzed using a multifactorial repeated-measures ANOVA with face category (three levels: friends 1 and 2 and partner) and hemisphere (two levels: right and left) as within-subject factors and gender as between-subjects factor. There was no main effect of face category and none of the interactions including face category (*face category x hemisphere, face category x gender, face category x hemisphere x gender*) reached significance; therefore these results are not reported in detail. There was a significant effect of hemisphere and the *hemisphere x gender* interaction for the M170 amplitude; for further analysis magnetic activity was averaged across all three face categories to perform a two-way mixed ANOVA with hemisphere as within-subject factor and gender as between subject factor on M100 and M170 amplitude and latency as well as dipole strength in both “free” and “fixed” condition. Significant main effects or interactions (*hemisphere x gender*) were further analyzed by post-hoc comparisons using independent samples t-tests for gender-comparisons and pairwise t-tests for comparisons between hemispheres; significance threshold was set to p<0.05. Statistical analysis was made using IBM™ SPSS™ Statistics Version 20.

## Results

**Figure 1:**
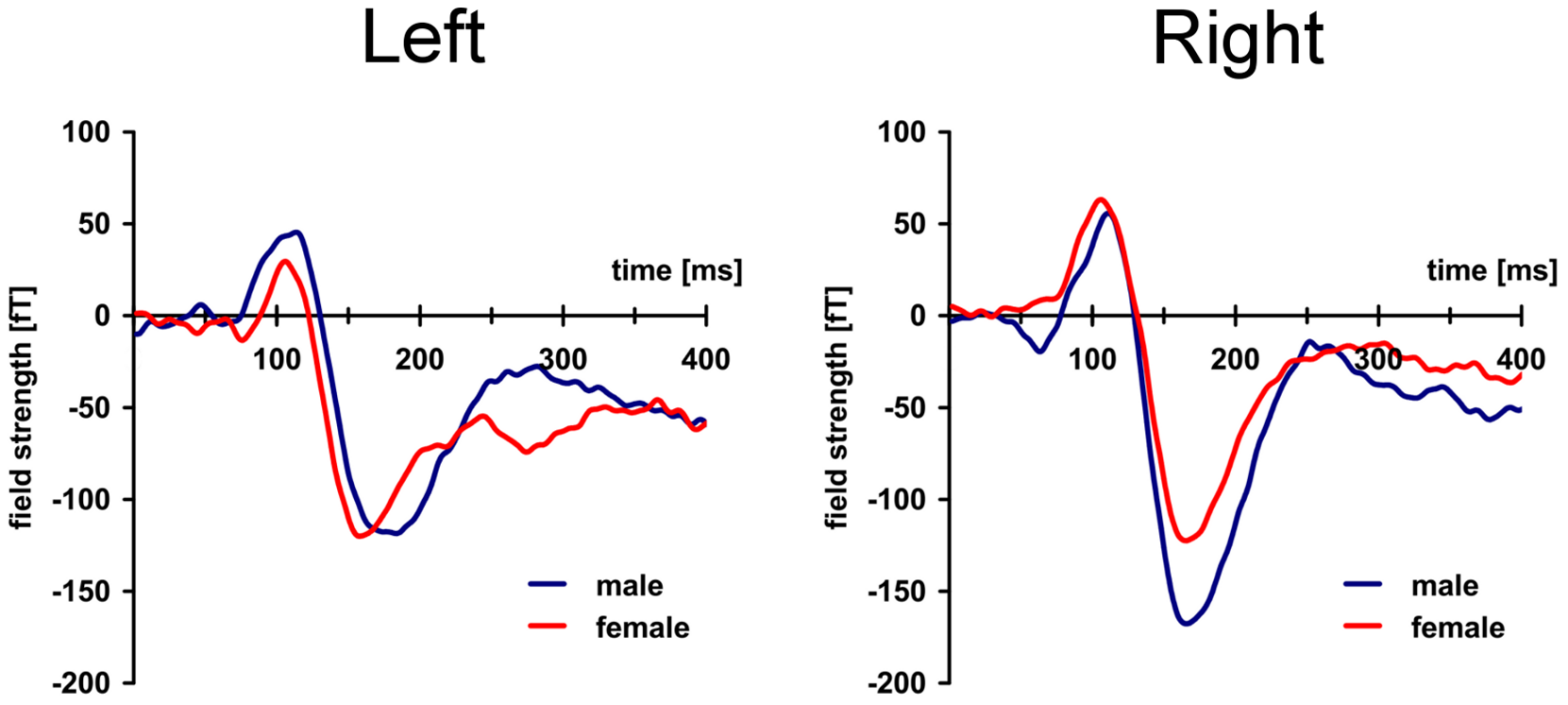
Sex-difference in hemispheric organization during face processing: Magnetic activity in sensor-clusters over left and right hemisphere averaged across 13 female (red) and 13 male (blue) subjects. M170 amplitude is larger in the right hemisphere in men without laterality effects in female participants. Note that M170-amplitudes were inverted to make comparisons possible.

**Figure 2:**
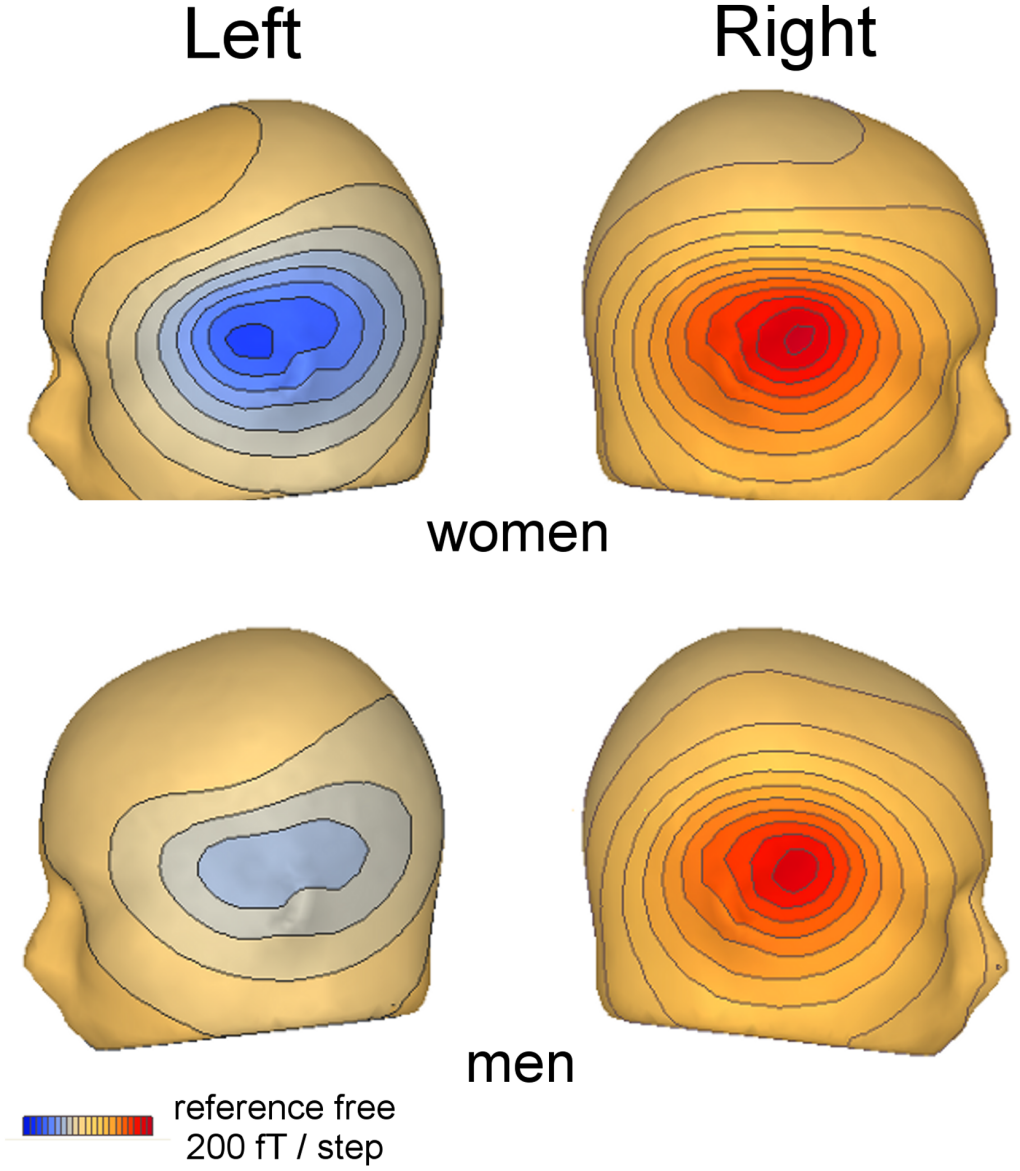
Field topography corresponding to the M170 peak-amplitude in each hemisphere and for men and women separately (time point of maximum is indicated as inset).

Grand average waveforms of magnetic activity recorded in sensor-clusters over both hemispheres between 0–400 ms are displayed for men and women each in Fig. 1, showing a M100 peaking at 108 ms (right) and 110 ms (left) and the M170-component with mean peak latencies of 169 ms (right) and 165 ms (left). Inspection shows a sex difference, i.e. an increased M170 amplitude in the right hemisphere in male participants. Fig. 2 shows the field topography of the M170 component also revealing a different degree of lateralized activity between genders.

### Amplitude and Latency of Early Event-Related Fields (ERFs)

A two-way mixed ANOVA calculated for the M170-amplitude yielded a significant main effect for hemisphere (F 1,24 = 7.425; p = 0.01*) and significant interaction of *gender x hemisphere* (F 1,24 = 10.795; p = 0.003**) without significant effects of between-subject comparison (p = 0.26). Pairwise comparisons of M170 amplitude shows that the M170-component was larger in the right compared to the left hemisphere (p = 0.029*). Further analysis of the interaction of *gender x hemisphere* regarding the M170-component revealed that the M170 was right-lateralized in men (p = 0.003**) without a significant difference of M170 amplitude between hemispheres in women (p = 0.65). A direct comparison between genders revealed an increased M170 amplitude in the right hemisphere in men as compared to women (p = 0.01*) without a difference for the left hemisphere (p = 0.81). There were no significant main effects or interactions for M100-amplitude or latencies of M100 and M170.

### M170 Dipole Localization and Strength

Dipole localization in the “free” condition was determined within the M170-timewindow in 24 individuals (13 male/11 female) and yielded Talairach-coordinates (x = ±23.3; y = −51.3; z = 2.4) resembling the localization of the FG in the “fixed” condition (x = ±29.0; y = −62.0; z = −15.0) unless for a different (inferior-superior) z-coordinate. Dipole localization in the “free condition” did not differ significantly (x-coordinate: p = 0.14; y-coordinate: p = 0.44; z-coordinate: p = 0.67) between men (x = ±21.1; y = −46.6; z = 0.5) and women (x = ±25.9; y = −57.2; z = 4.6).

ANOVAs performed on dipole strength in the M170-timewindow yielded a significant main effect of hemisphere in both “fixed” (F 1,24 = 6.189; p = 0.02*) and “free” conditions (F 1,22 = 6.448; p = 0.01*) as well as a significant interaction of *hemisphere x gender* again both in the “fixed” (F 1,24 = 6.189; p = 0.03*) as well as the “free” condition (F 1,22 = 11.946; p = 0.002**). Paired-comparisons for all subjects indicated enhanced dipole strength in the right as compared to the left hemisphere in the “free” and “fixed” condition (both comparisons: p = 0.03*). Further analysis of the *hemisphere x gender* interaction revealed a similar pattern as observed for M170-amplitude: When analyzed separately for men and women each activity was right-lateralized only in male subjects both in the “free” (p = 0.0001**) and “fixed” condition (p = 0.005**), whereas there were no differences between hemispheres in women (“free”: p = 0.61; “fixed”: p = 0.87).

### Results - Summary

Neither amplitude nor latency of early face-specific ERFs (M100, M170) was modulated by face category. Both the M170 amplitude as well as calculated dipole strength in the M170-timewindow revealed a pattern of right-lateralized face processing in men and bilateral activation in women. Dipole localization obtained in the “free” condition did not differ between genders resembles previous studies on the FG based on neuroimaging and EEG/MEG recordings [2], [18]. However, there was considerable difference concerning the (inferior-superior) z-coordinate. Essentially, both the analysis of M170-amplitude and dipole strength in the M170 time window yielded similar results indicating asymmetric involvement of face-responsive regions, as indicated by source localization most notably the FG in men with no such difference in female participants. Results of all post-hoc comparisons for significant main effects or interactions indicated by ANOVA for the M170 (amplitude and dipole strength) are shown in Tab. 1.

**Table 1:** Lateralization of M170 amplitude and dipole strength.

	All	Men	Women
	Left/Right	Left/Right	Left/Right
M170 Amplitude [fT]	−145.2/−176.3	−141.3/−209.8	−149.1/−142.7
	t = –2.310	t = –3.775	t = 0.463
	p = 0.029*	p = 0.003**	p = 0.65
Dipole strength [nAm]	33.1/46.1	22.6/50.0	45.6/41.4
“free” condition	t = 2.335	t = 5.624	t = –0.516
	p = 0.029*	p = 0.0001**	p = 0.61
Dipole strength [nAm]	26.1/34.1	17.3/35.5	34.9/35.6
“fixed” condition	t = 2.307	t = 3.391	t = 0.165
	p = 0.03*	p = 0.005*	p = 0.87

Post-Hoc comparisons of significant main effects or interactions indicated by ANOVAs for M170 amplitude and dipole strength obtained within the M170-timewindow. Note that analysis of dipole strength in the “free” condition is based on 11 female subjects because no plausible solution could be obtained in 2 participants; all other analyses include 13 subjects of each gender. Comparisons between hemispheres were based on paired-sample t-tests. p-values<0.05 are indicated by *, p-values <0.01 by **.

## Discussion

Our results of a larger right-lateralized M170 amplitude in men as well as asymmetric strength of the underlying dipole is in line with previous work describing lateralized face processing in men compared to bilateral coding of faces in women [7].

The present study using MEG combined with dipole-localization makes an important additional contribution, showing that the hemispherical difference in source strength at 170 ms as well as M170 amplitude have to be due to differential involvement of the FG in men and women. This conclusion is supported in particular by concurrent results obtained in the analysis of M170 amplitude as well as dipole strength within the timewindow of the M170-component revealing asymmetric activation for men as compared to bilateral activation in women showing no difference between hemispheres. A similar pattern regarding amplitude of the N170-component of the ERP was observed in the investigation by Proverbio et al. [7], however source localization using LORETA reconstruction resulted in rather extended bilateral activity maps with left asymmetry in women and a right asymmetry in men. This limits inferences about circumscribed neural structures such as FG underlying this asymmetry whereas the localization method applied to MEG-data in our study follows a different mathematical approach with point-like source localization. This also has limitations because one can not draw conclusions about the three-dimensional extent of the sources but in the present case this “limitation” turns out to be very powerful: Using a single dipole model for each hemisphere the group mean sources are localized in the FG and this localization does not differ between men and women. This is not compatible with the interpretation that sources outside the FG significantly contribute to differential hemispheric processing as observed here because this should have resulted in different source locations between sexes. In general the M170 is assumed to primarily originate from sources in the FG because their dominant tangential orientation is preferentially picked up by MEG whereas EEG is sensitive to sources of tangential as well as radial orientation, outside FG such as the superior temporal sulcus (STS) [17]. This is one reason for the heterogeneity of results of studies that modelled the N170 source whereas a FG source was consistently found across studies for the M170 (for a detailed discussion see [18], [19]).

Source localization obtained in the “free” condition and the “fixed” condition based on Talairach coordinates of the FG yielded similar results unless for a more superior z-coordinate in the “free” condition, implicating another source outside the FG such as the STS. This can be interpreted in the way that passive viewing of familiar faces might engage face responsive regions in FG (and possibly STS) in a distinct way than age classification tasks employed in the study by Deffke et al. [18] which we based our “fixed” condition on, given that the STS is activated during a variety of tasks relating to social cognition [21]. In addition, recent studies have indicated interactions between the FG and STS during face processing [22] which is compatible with studies highlighting the role of functional connectivity between face-responsive neural structures, e.g. [23]. However, it should also be noted that ICA-correction and removal of cardiac artefacts typically recorded by the most inferior MEG-sensors have been reported to result in shifted z-coordinates and more superior source localizations [18].

How does our finding fit into the body of existing literature? The right hemisphere has been implicated in global compared to local processing [24]. Furthermore, studies of acquired prosopagnosia [25] and those with infants suffering from congenital cataract [3] suggest that right occipito-temporal structures and particularly the FG [26], [27] subserve configural/holistic face processing. Accordingly the lateralization effect observed in men here can be interpreted to indicate a bias towards holistic processing of opposite sex faces, considering that both strategies are employed flexibly during face processing [28], [29].

It has been demonstrated that women spend more time looking at the eyes whereas men pay more attention to central parts of faces (nose, mouth), indicating a more globally oriented processing [30] based on a centred viewpoint compared to more fixations at the eyes in a local strategy [28]. Moreover, men exhibit greater interest in opposite-sex faces than women [31] and value attractiveness higher than women [32], [33] which is reflected in stronger recruitment of reward-related areas in men viewing attractive female faces [34]. The processing and appreciation of certain social aspects of faces and essentially attractiveness arguably requires more holistic processing [35], [36]. Also, viewing female faces increases FG activation in men regardless of task whereas the activity when viewing male faces depends if they are targets or non-target stimuli suggesting that men perceive female faces as natural targets [37].

This finds support in the view that aesthetic judgements of faces may be enhanced by some variant features (e.g. smile, [38]) but is particularly based on invariant facial features such as averageness, symmetry and sexual dimorphism [39]. According to the model of Haxby et al. invariant features are processed in the (lateral) FG [1]. Thus, the FG but not STS has been shown to be involved in both explicit and implicit processing of attractiveness [40] and lesions affecting the FG result in impaired judgements of facial identity as well as attractiveness in patients with acquired prosopagnosia [41].

Given the aforementioned sex-differences in visual strategy and interest in opposite-sex faces these studies account for a differential involvement of the FG in men as observed here yet it also raises questions about possible contributions of brain structures implicated in face evaluation and emotion processing. We did not find a modulation of early face processing by emotional and social context which is in accordance with previous investigations consistently showing that ERPs do not differentiate faces of different categories, e.g. familiarity (partner, family members, unfamiliar) or attractiveness before 200–300 ms in passive viewing tasks [42]–[44] (for a discussion also see [45]). Nevertheless it is an intriguing question how our result relates to sex-differences in lateralization of amygdala activation during the processing of emotional stimuli (for a recent meta-analysis see [46]). There is compelling evidence for a modulation of FG activity through functional connectivity with the amygdala during perception of fearful faces [47], for review see [48]. But there is no indication that these interactions would influence the N170/M170 response considering that intracranial recordings do not show modulation of amygdala activity by emotional content before 200 ms post stimulus [49]–[52] (for review see also [53]). Note that personally familiar faces are associated with decreased amygdala activity [54] in BOLD-fMRI investigations bringing little information about the timing of neural activity due to the low temporal resolution of this method.

Finally some restrictions of the present study have to be mentioned: It was conducted with heterosexual men and women perceiving opposite sex faces with no explicit task requirement. Future studies have to include same sex faces and have to address the important question whether the lateralization effect observed here depends on specific task requirements, i.e. local (featural) vs. global (holistic/configural).

Also interactions of biological sex and psychological gender indentity have to be taken into account, since a relationship between lateralisation patterns and psychological masculinity in men has been demonstrated [55]. Likewise, the influence of “gender enculturation” [56] on social cognition has to be considered to avoid premature and inconsistent conclusions about hard-wired differences between male and female brains. It has been suggested, that sex-differences observed in cognitive functions may be linked to differences in brain anatomy, e.g. [57], yet some of these findings are found to be inconsistent, hence the relationship between sex-differences in cognition and anatomy remains an issue that is rather unsettled [56].

### Conclusions

Our results specify results from previous studies suggesting a sex-difference in hemispheric processing of faces. This effect has to be taken into account if lateralisation effects of face processing are described, in particular if they regard activation of the FG. Future research is necessary to elucidate the impact of important factors on this asymmetric representation, e.g. task or psychological gender identity.
